# Bile microbiota in gallbladder stones and its association with *Helicobacter pylori*: a systematic review and meta-analysis

**DOI:** 10.3389/fmicb.2025.1707225

**Published:** 2026-01-13

**Authors:** Hao Li, Huiyao Zhang, Wenyu Wu, Tingting Zhou, Xiaoxiao Chen, Yang Yang, Wei Wei

**Affiliations:** 1 Wangjing Hospital, China Academy of Chinese Medical Sciences, Beijing, China; 2 Guangzhou University of Chinese Medicine, Guangzhou, China

**Keywords:** bile microbiota, gallbladder stones, *Helicobacter pylori*, meta-analysis, systematic review

## Abstract

**Introduction:**

Gallbladder stones (GS) is a prevalent gallstone disease. Recent studies indicate that bile microbiota dysregulation may contribute to their pathogenesis. However, the specific microbial alterations and their differences from gut microbiota patterns remain unclear. This study aimed to systematically evaluate the association between bile microbiota composition and GS.

**Methods:**

Eligible studies comparing bile microbiota profiles between patients with GS and non-GS controls were retrieved from eight databases. Data on *α*- and *β*-diversity and microbial composition at the phylum and genus levels were extracted and synthesized.

**Results:**

For *α*-diversity, the abundance-based coverage estimator (ACE) index was higher in patients with GS compared with controls (SMD = 0.55, 95% confidence interval [CI]; 0.23–0.87), whereas the Chao1 (SMD = 0.51, 95% CI; −0.03–1.05) and observed species (SMD = 0.58, 95% CI; −0.03–1.19) showed positive but non-significant differences. The Simpson index was significantly higher in patients with GS (SMD = 0.49, 95% CI; 0.02–0.96). The Shannon index showed no overall difference (SMD = 0.03, 95% CI; −0.51–0.56), but was significantly decreased in the “GS vs. healthy controls (HC)” subgroup (SMD = −0.48, 95% CI; −0.94–-0.02). Analysis of *β*-diversity revealed that the bile microbiota of patients with GS differed significantly from that of control groups, although a few studies reported no significant differences. At the phylum level, patients with GS consistently exhibited increased Firmicutes and decreased Proteobacteria. At the genus level, enrichment of pathogenic taxa, such as *Escherichia–Shigella* and *Streptococcus,* was observed, whereas *Helicobacter* was elevated in only one study, indicating that its association with GS may be context-dependent and warrants further investigation.

**Conclusion:**

GS is associated with bile microbiota dysbiosis, characterized by increased richness and potential overrepresentation of dominant taxa. These alterations differ from gut microbiota patterns in GS, suggesting a unique role of bile microbiota in GS pathogenesis.

**Systematic review registration:**

https://www.crd.york.ac.uk/prospero/, identifier CRD420251028569.

## 1 Introduction

Gallstone disease, a common and prevalent biliary tract disorder worldwide, has high incidence and recurrent acute episodes, imposing a substantial burden on public-health systems. A global epidemiological analysis estimated that approximately 6% of the world population was affected by gallstone disease (Wang X. et al., 2024). According to an analysis of the 2021 Global Burden of Disease study (Dai et al., 2025), the age-standardized incidence rate exhibited a declining trend, but the absolute number of gallbladder and biliary tract disease cases increased by 60.11% globally, owing to population growth and aging. Notably, the disease burden exhibits substantial regional heterogeneity. Gallbladder stones (GS), the most common type of gallstone disease, are classified into cholesterol, mixed, and pigment stones. Its pathogenesis is multifactorial, with stone formation resulting from any disturbance in the cholesterol-to-bile acid phospholipid ratio or bile stasis (Lammert et al., 2016; Reshetnyak, 2012). Although laparoscopic cholecystectomy (van de Graaf et al., 2018) remains the standard treatment for symptomatic or complicated cases, postoperative complications, such as bile leakage, common bile duct injury, and postcholecystectomy syndrome, remain significant clinical challenges. Accordingly, preventive strategies, mechanistic understanding of pathogenesis, and therapeutic interventions for GS are urgently needed.

However, the traditional “5Fs” risk factors (fair, fat, female, fertile, and forty) (Bass et al., 2013) and classical mechanisms, such as imbalances in bile composition and gallbladder dysmotility, fail to fully account for the geographic, ethnic, age-related, and sex-based variations observed in gallstone disease. Recently, the emergence of the “gut–biliary axis” concept has attracted attention to the role of the gut microbiota in gallstone pathogenesis, particularly through its regulation of bile acid metabolism, cholesterol homeostasis, and gallbladder motility (Liu et al., 2025; Wang et al., 2025). Additionally, bacterial colonization and biofilm formation in the gallbladder contribute to gallstone formation by inducing chronic inflammation, altering bile composition, and promoting cholesterol crystallization (Wang D. et al., 2024). In a study involving 29 patients with gallstones and chronic cholecystitis (Zhang et al., 2024), the microbial *α*-diversity in bile samples was higher than that in gallstone samples. Integrated proteomic, metaproteomic, and NCBI-derived 16S rRNA profiling of human bile revealed a rich spectrum of phyla (Yang et al., 2023). Recent studies show that the biliary bacteria may originate from the intestine through backward movement via the duodenum (Wang et al., 2017; Ye et al., 2016; Zhang et al., 2025). Therefore, several studies have speculated that *Helicobacter pylori* may contribute to GS formation (Wang et al., 2021; Zhang et al., 2015). However, current evidence is largely restricted to association analyses of gut microbiota or indirect inferences from Mendelian randomization studies (Zhou et al., 2025). Systematic and comparative investigations of the bile microbiota remain limited, leaving the potential link between *H. pylori* and gallstones largely speculative. Moreover, current some studies are limited by small sample sizes, different sequencing platforms, and the lack of standardized data integration (Li et al., 2025).

Therefore, we systematically searched eight Chinese and English databases (until July 2025) and conducted the first meta-analysis comparing the bile microbiota between patients with GS and controls. Subgroup analyses were performed to identify potential sources of heterogeneity, and the differences between patients with GS and controls were further summarized. The study was registered with PROSPERO (CRD420251028569) before initiation.

## 2 Materials and methods

### 2.1 Search strategy

In accordance with Cochrane Handbook for Systematic Reviews of Interventions (Cumpston et al., 2019), we systematically searched PubMed, Web of Science, Cochrane Library, Embase, CNKI, Wanfang, VIP, and CBM between January 1, 2006 and July 3, 2025. The search combined Medical Subject Headings (MeSH) terms with free-text terms. The MeSH terms included “Cholecystolithiasis” and “Microbiota.” All procedures adhered to the PRISMA 2020 statement (Page et al., 2021). An example of the search strategy is provided in Supplementary Table 1.

### 2.2 Study selection criteria

The study was designed in accordance with the PICO (Population, Intervention, Comparison, Outcome) framework to investigate the association between cholecystolithiasis and the microbiota. Eligible studies included all clinical research published in Chinese or English that investigated microbiota dysbiosis in relation to cholecystolithiasis or gallstones. Trial type was not restricted. The target population comprised patients diagnosed with GS, with no restrictions on race, nationality, or disease duration. Control groups comprised individuals without cholecystolithiasis, excluding those with gallbladder cancer.

Studies were excluded if they (1) lacked a clearly defined design or methodology, (2) failed to provide extractable outcome data, (3) duplicated previously published data, (4) lacked full-text availability, or (5) had a sample size insufficient for meaningful statistical analysis. Animal studies, *in vitro* experiments, case reports, review articles, meta-analyses, and letters to the editor were excluded.

### 2.3 Outcome measures

Eligible studies were required to report at least one of the following outcomes: *α*-diversity, *β*-diversity, or differences in microbial composition.

### 2.4 Data extraction

During data extraction, two researchers (Hao Li and Huiyao Zhang) independently assessed and extracted information from studies that met the inclusion criteria. The extracted data included the first author’s name, publication year, study region, disease type of the control group, sample size, participant age, detection method, sequencing fragment, α- and β-diversity indices, and microbial composition. Control groups were categorized as healthy controls (HC), bile duct stones controls (BDS), or other controls, such as cholecystitis and gallbladder polyps. For studies that presented data in graphical formats, the reported five-number (minimum, first quartile, median, third quartile, and maximum) were extracted from the figures using GetData 2.20. After estimating the mean and standard deviation using an online platform^1^, any indicator whose data exhibited significant skewness in all three examined scenarios was excluded from the meta-analysis. Microbial composition at the taxonomic level was recorded. Disagreements between the two researchers were resolved through discussion or, if needed, consultation with a third reviewer to ensure the reliability.

### 2.5 Quality assessment

Two researchers (Hao Li and Huiyao Zhang) independently evaluated the methodological quality of the included studies using the Newcastle–Ottawa Scale (NOS) (Stang, 2010), a tool specifically designed for assessing observational studies and widely used in systematic reviews and meta-analyses to evaluate the risk of bias (Wang et al., 2022). The NOS assesses three domains and assigns a score from 0 to 9.

The risk of bias in each included study was assessed using the revised Risk of Bias Assessment Tool for Nonrandomized Studies (RoBANS 2). Based on the official RoBANS 2 criteria, eight domains were assessed and categorized as “low risk,” “unclear risk,” or “high risk.” Discrepancies were resolved by discussion or input from a third reviewer, if needed.

## 3 Results

### 3.1 Study selection, characteristics, and quality of the studies

**Figure 1:**
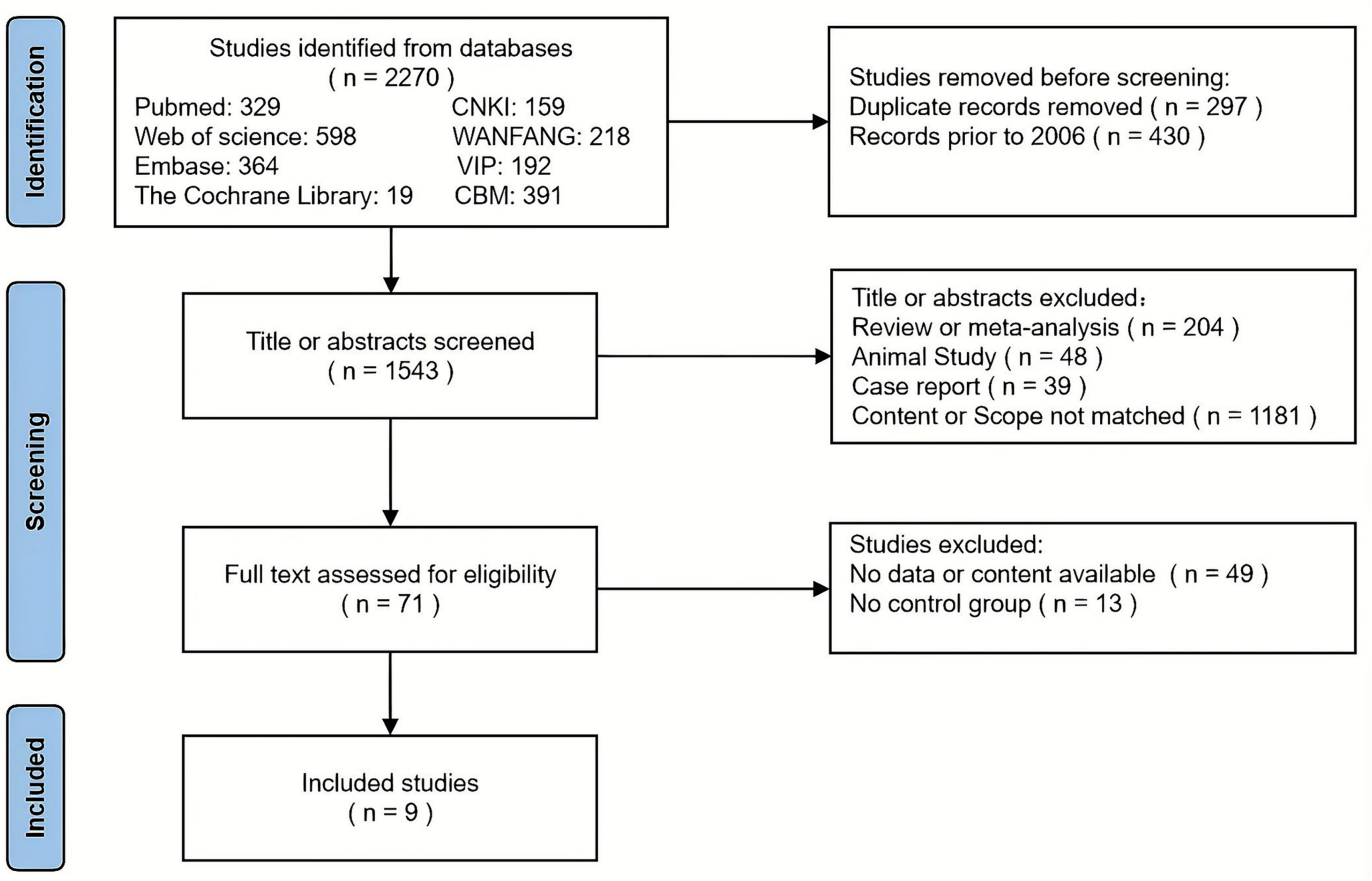
Retrieval flow diagram.

The study selection process was conducted in accordance with the PRISMA 2020 (Figure 1). Among 2,270 records retrieved from eight databases, 430 records published before 2006 were initially excluded. After removing 297 duplicates, 1,543 records remained. Two reviewers independently screened the titles and abstracts against predefined inclusion and exclusion criteria, excluding 1,472 irrelevant or non-compliant articles. Consequently, 71 articles underwent full-text evaluation. Subsequently, 49 articles were excluded for “no usable data,” and 13 were excluded for “no appropriate control group,” resulting in nine studies eligible for the meta-analysis (Table 1). All nine studies included in this review employed high-throughput sequencing of the 16S rRNA. Regarding methodological quality, the NOS scores ranged from 5 to 8 (Supplementary Table 2), and the results of the RoBANS 2 are presented in Supplementary Table 3.

**Table 1:** Basic characteristics of the nine included studies.

Study	Sequencing	Clustering	Similarity	Patient group	Control group	Source of sample	Sample size	Age
Yu et al. (2024a)	16S rRNA	OTU	97%	Gallstones	Donors without hepatobiliary disorder	Gallbladder and bile	G:6 C:6	–
Park and Park (2024)	16S rRNA V3–V4	OTU	97%	Cholecystolithiasis	Choledocholithiasis	Bile	G:11 C:17	G:59 (30–81) C:67 (27–88)
Cai et al. (2023)	16S rRNA V3–V4	ASV	–	Cholecystolithiasis	Donors without hepatobiliary disorder	Bile	G:15 C:8	G:39.71 ± 12.37 C:32.37 ± 10.34
Wang (2023)	16S rRNA V3–V4	OTU	97%	Gallbladder cholesterol stones	Gallbladder cholesterol polyps	Bile	G:12 C:10	G:56.75 ± 5.24 C:41.50 ± 3.77
Lu (2023)	16S rRNA V3–V4	OTU	97%	Cholecystolithiasis	Gallbladder polyps	Bile	G:40 C:9	G:39.5 (30.5–46) C:36 (27.5–48.0)
Du et al. (2022)	16S rRNA VI–V2	OTU	97%	Gallbladder cholesterol stones	Cholecystitis, gallbladder polyps	Bile	G:42 C:40	G:47.8 ± 10.2 C:45.3 ± 4.9
Liao (2021)	16S rRNA V3–V4	OTU	97%	Cholecystolithiasis	Liver cyst, liver hemangioma	Bile	G:31 C:9	G:51 ± 15 C:46 ± 10
Feng (2021)	16S rRNA	OTU	97%	Cholecystolithiasis, bile duct stones	Bile duct cancer, pancreatic cancer, gallbladder polyps	Bile	G:52 C:11	G:55.19 ± 16.63 C:60.64 ± 12.91
Molinero et al. (2019)	16S rRNA V3	OTU	100%	Cholelithiasis	Donors without hepatobiliary disorder	Bile	G:14 C:13	G:27–73 C:37–79

G, patients with gallstone; C, control group.

### 3.2 Synthesis of *α*-diversity

α-diversity is assessed using measures of species richness, species evenness, or indices that synthesize both dimensions (Jovel et al., 2016; Lozupone and Knight, 2008; Whittaker, 1972). Eight of the nine studies reported α-diversity data. Of these, two studies (Feng, 2021; Cai et al., 2023) included control groups subdivided according to different classification diseases. We evaluated α-diversity using observed species, Chao1, ACE, Shannon, and Simpson. Compared with the observed species index, richness estimators, such as Chao1 and ACE, account for rare taxa (Chao, 1984). Community diversity, integrating richness and evenness, is commonly assessed using the Shannon and Simpson indices, which provide insights into the structural complexity (Simpson, 1949).

**Figure 2:**
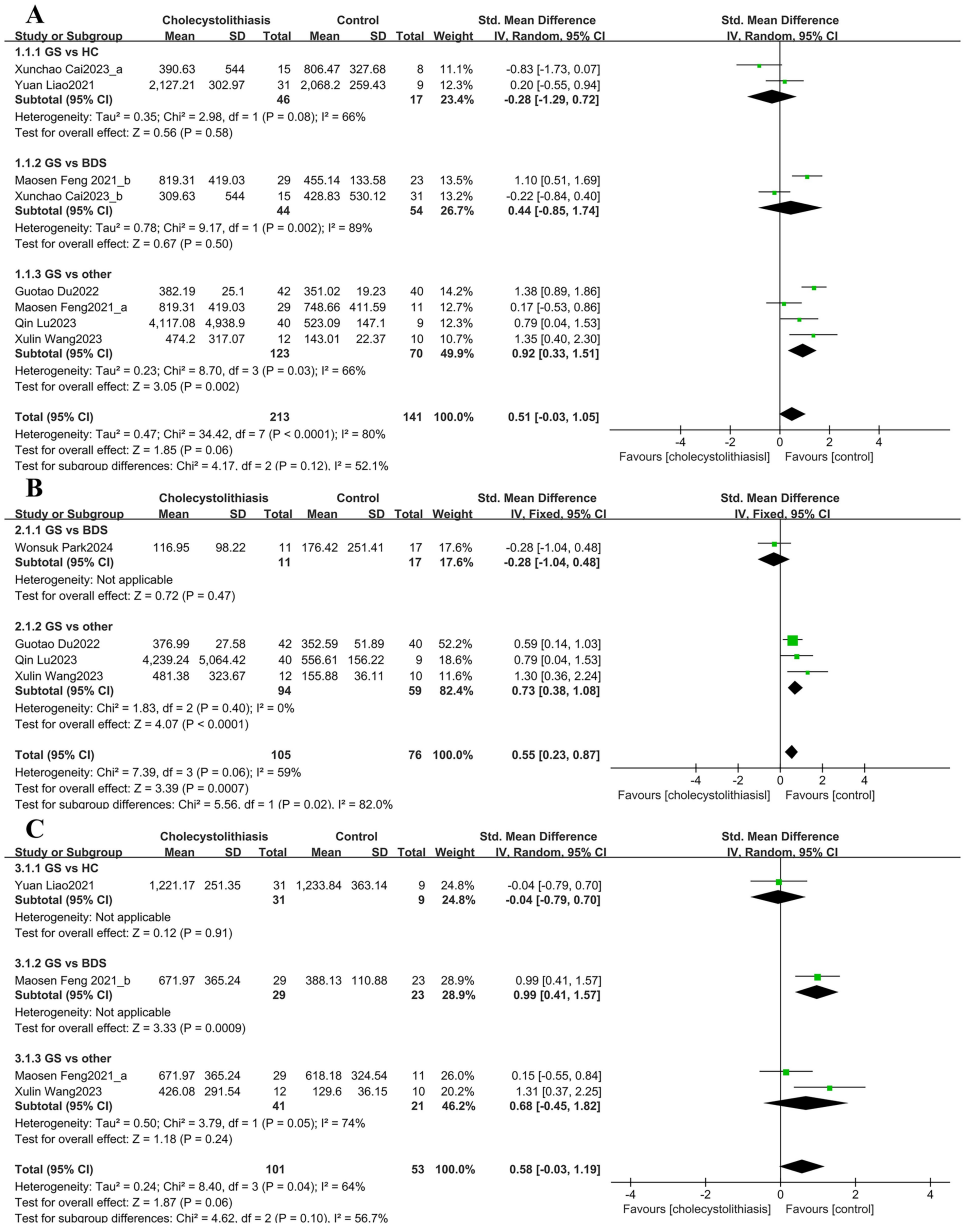
Forest plots of *α*-diversity indices (Chao1, ACE, and observed species) of the bile microbiota in patients with GS compared with various controls. Panels show **(A)** Chao1, **(B)** ACE, and **(C)** Observed species. A random-effects model was used for panels **(A,C)**, and a fixed-effect model was used for panel **(B)**.

Among the richness estimation indices, six studies were included for the Chao1 index. The pooled analysis revealed substantial heterogeneity (I^2^ = 80.00%, *p* < 0.0001); therefore, a random-effects model was applied (Figure 2A). The difference between groups was not statistically significant (SMD = 0.51, 95% CI; −0.03–1.05). Subgroup analysis of the Chao1 index revealed a significant increase in the “GS vs. other” comparison (SMD = 0.92, 95% CI; 0.33–1.51), whereas no significant differences were detected in other subgroup comparisons. For the ACE index, four studies were included, comprising 105 patients with GS and 76 controls (Figure 2B). The heterogeneity test showed moderate heterogeneity (I^2^ = 59.0%, *p* = 0.06). The pooled results demonstrated a significantly higher ACE index in the GS group (SMD = 0.55, 95% CI; 0.23–0.87). Three studies reported the observed species index (Figure 2C). Owing to heterogeneity, a random-effects model was applied, and the pooled results indicated no significant difference between groups (SMD = 0.58, 95% CI; −0.03–1.19).

**Figure 3:**
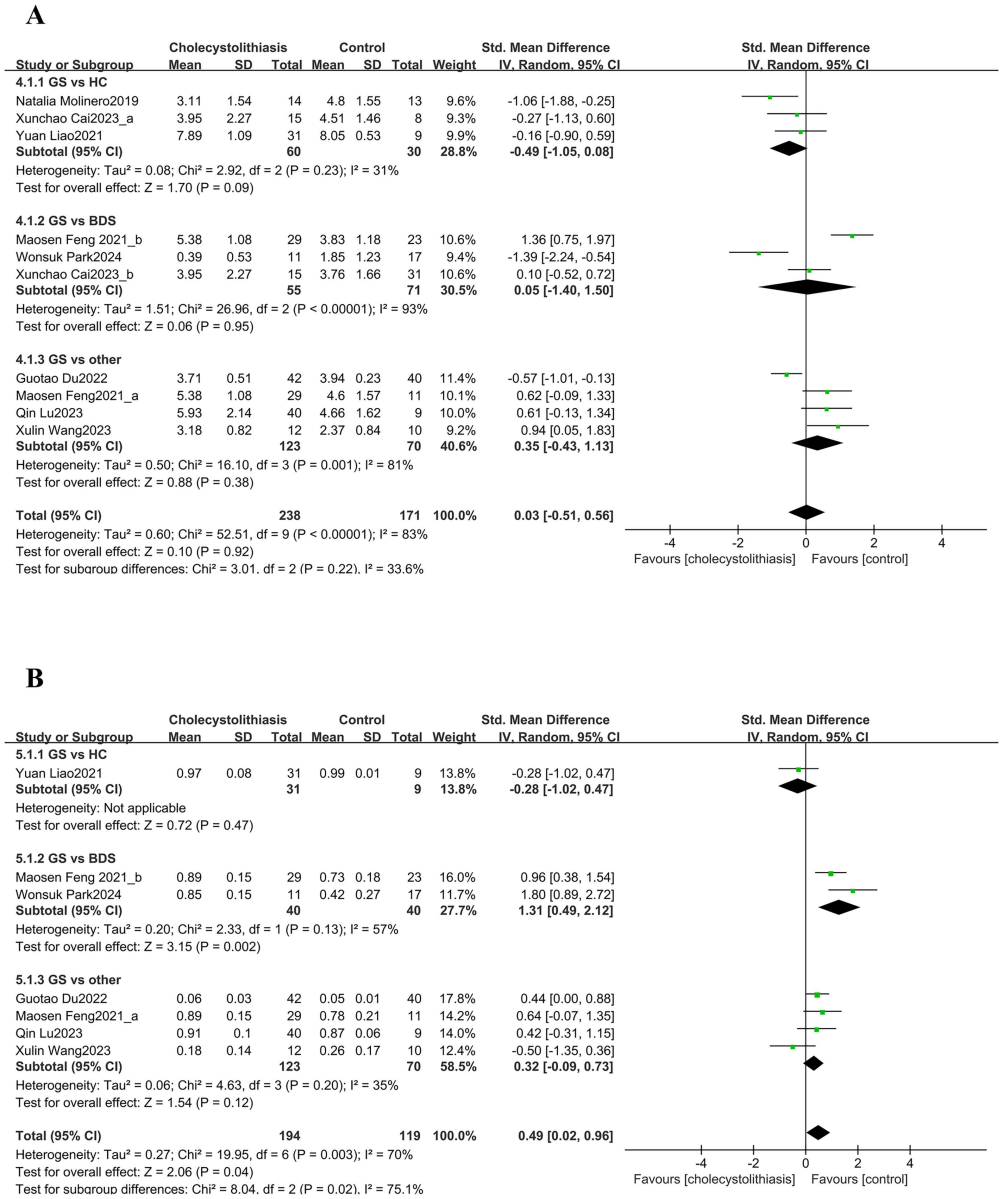
Forest plots of α-diversity indices (Shannon and Simpson) of the bile microbiota in patients with GS compared with various controls. Panels show **(A)** Shannon and **(B)** Simpson. Both panels were generated using a random-effects model.

Among the richness and evenness indices, eight studies evaluated the Shannon index. The pooled analysis indicated significant heterogeneity (I^2^ = 83.0%, *p* < 0.00001). Therefore, a random-effects model was used (Figure 3A). Overall, no significant difference was observed (SMD = 0.03, 95% CI; −0.51–0.56). In the subgroup analysis, no significant heterogeneity was detected in the “GS vs. HC” comparison, and the fixed-effects model indicated a significantly lower Shannon index in the gallstone group (SMD = −0.48, 95% CI; −0.94–-0.02) (Supplementary Figure 1). The pooled analysis of the Simpson index revealed significant heterogeneity across studies (I^2^ = 70.0%, *p* = 0.003); thus, a random-effects model was used (Figure 3B). Overall, the GS group showed a significantly higher Simpson index (SMD = 0.49, 95% CI; 0.02–0.96). Further subgroup analyses revealed no significant heterogeneity in either the “GS vs. BDS” or “GS vs. other” comparisons. Using fixed-effects models, the Simpson index remained significant different in the gallstone group (GS vs. BDS: SMD = 1.20, 95% CI; 0.71–1.69; GS vs. other: SMD = 0.35, 95% CI; 0.04–0.66) (Supplementary Figure 2). If < 10 studies were available, funnel plots, or statistical tests were not recommended for assessing publication bias.

### 3.3 Synthesis of *β*-diversity

Among the nine included studies, eight evaluated β-diversity (Table 2), primarily through principal coordinate analysis (PCoA) based on weighted and unweighted UniFrac distances. In comparisons between patients with GS and HC, three studies (Liao, 2021; Molinero et al., 2019; Yu et al., 2024a) reported significant differences in microbial community structure, whereas one study (Cai et al., 2023) found no such difference. Similarly, in comparisons between patients with GS and those with BDS, two studies (Feng, 2021; Park and Park, 2024) identified significant differences (*p* < 0.05), whereas the study (Cai et al., 2023) again observed no significant difference. Furthermore, two studies comparing patients with GS with other controls (Lu, 2023; Wang, 2023) consistently showed significant differences (*p* < 0.05). Overall, current findings indicate inconsistent evidence regarding β-diversity differences between patients with GS and various control groups, highlighting the need for further research and standardized analytical approaches to improve reproducibility and comparability.

**Table 2:** *β*-diversity indices reported in the eight included studies.

Study	*β*-diversity	Findings	Statistic value
Yu et al. (2024a)	PCoA based on weighted Unifrac dissimilarity	A significant difference in gallbladder mucosal bacteria between GS and HC	*p* < 0.05
Park and Park (2024)	PCoA employing generalized UniFrac and UniFrac metrics	A significant difference in the bile microbial composition between GS and BDS	*p* < 0.05
Cai et al. (2023)	PCoA of weighted UniFrac distances	No significant difference among GS, HC and BDS	NR
Wang (2023)	PCoA of weighted Unifrac distances and unweighted Unifrac distances	A significant difference in the bile microbial composition between GS and other controls	*p* = 0.001, *p* < 0.001
Lu (2023)	PCoA of weighted Unifrac distances and unweighted Unifrac distances	A significant difference in the bile microbial composition between GS and other controls	*p* = 0.019, *p* = 0.029
Liao (2021)	PCoA	A significant difference in the bile microbial composition between GS and HC	NR
Feng (2021)	PCoA of weighted Unifrac distances	A significant difference in the bile microbial composition between GS and BDS	*p* = 0.001
Molinero et al. (2019)	PCoA of weighted UniFrac distances	A significant difference in the bile microbial composition between GS and HC	*p* < 0.001

NR, not reported.

### 3.4 Differences in microbial composition

At the phylum level, nine studies reported microbiota alterations in patients with GS compared with controls, with the most consistent findings being increased Firmicutes in patients with GS and relatively higher Proteobacteria in controls (Cai et al., 2023; Lu, 2023; Park and Park, 2024; Wang, 2023). Actinobacteria, Bacteroidetes, and Fusobacteria exhibited variable patterns of increase and decrease in patients with GS compared with controls, reflecting inconsistency across studies (Cai et al., 2023; Du et al., 2022; Feng, 2021; Liao, 2021; Lu, 2023; Molinero et al., 2019; Park and Park, 2024; Wang, 2023). Among the four studies involving Bacteroidetes, three reported higher abundance in gallstone patients, excluding the study including controls with choledocholithiasis (Park and Park, 2024). Similar findings were observed for Verrucomicrobia (Park and Park, 2024; Wang, 2023; Yu et al., 2024a). Furthermore, certain less prevalent phyla, including Deferribacteres, Deinococcota, and Euryarchaeota, were reported only in individual studies, suggesting that their alterations may not be specific to GS (Table 3).

**Table 3:** Phylum-level differences in relative microbial abundance between gallstone patients and control groups.

Phylum	Yu et al. (2024a)	Park and Park (2024)	Cai et al. (2023)	Wang (2023)	Lu (2023)	Du et al. (2022)	Liao (2021)	Feng (2021)	Molinero et al. (2019)
Statistical differences	+	−	−	+	−	−	+	+	+
Proteobacteria		Higher (C)*	Higher (C)	Higher (C)	Higher (C)				
Firmicutes		Higher (G)	Higher (G)	Higher (G)	Higher (G)				
Actinobacteria		Higher (C)			Higher (G)			Higher (G)	
Bacteroidetes		Higher (C)	Higher (G)		Higher (G)				Higher (G)
Fusobacteria		Higher (C)		Higher (G)	Higher (C)	Higher (C)	Higher (G)		
Cyanobacteria					Higher (C)				
Verrucomicrobia	Higher (G)	Higher (C)		Higher (G)					
Gemmatimonadota	Higher (C)			Higher (G)					
Planctomycetes					Higher (C)				
Acidobacteriota	Higher (C)				Higher (G)				
Deferribacteres							Higher (G)		
Nitrospirae							Higher (C)		
Deinococcota	Higher (G)								
Euryarchaeota	Higher (G)								
Myxococcota	Higher (C)								

G, patients with gallstone; C, control group. Statistical differences are denoted by “+” in a study, the list includes only those phyla with statistical differences. If denoted by “–,” the phyla are qualitatively compared according to the textual descriptions and figures provided in the study, whereas “*” denotes that the study reports significant differences.

At the genus level, eight studies reported compositional alterations between patients with GS and controls (Supplementary Table 4) (Cai et al., 2023; Du et al., 2022; Feng, 2021; Lu, 2023; Molinero et al., 2019; Park and Park, 2024; Wang, 2023; Yu et al., 2024a). The most consistent finding was an increased abundance of *Lactobacillus*, *Escherichia-Shigella*, *Streptococcus*, and *Cupriavidus* in patients with GS, whereas genera, such as *Sphingomonas*, *Bradyrhizobium*, *Pseudomonas*, and *Acidibacter* were more often higher in controls. Notably, *Helicobacter* was uniquely reported as increased in patients in the study (Yu et al., 2024a), indicating possible context-specific alterations. Genera, such as *Ralstonia*, *Vibrio*, and *Bacteroides,* showed variable patterns of increase and decrease in patients with GS compared with controls, reflecting inconsistency across studies. Other genera, including *Neisseria*, *Atopobium*, *Oribacterium*, and *Acinetobacter*, were observed exclusively in single studies, suggesting that their alterations may not be consistently associated with GS.

## 4 Discussion

**Figure 4:**
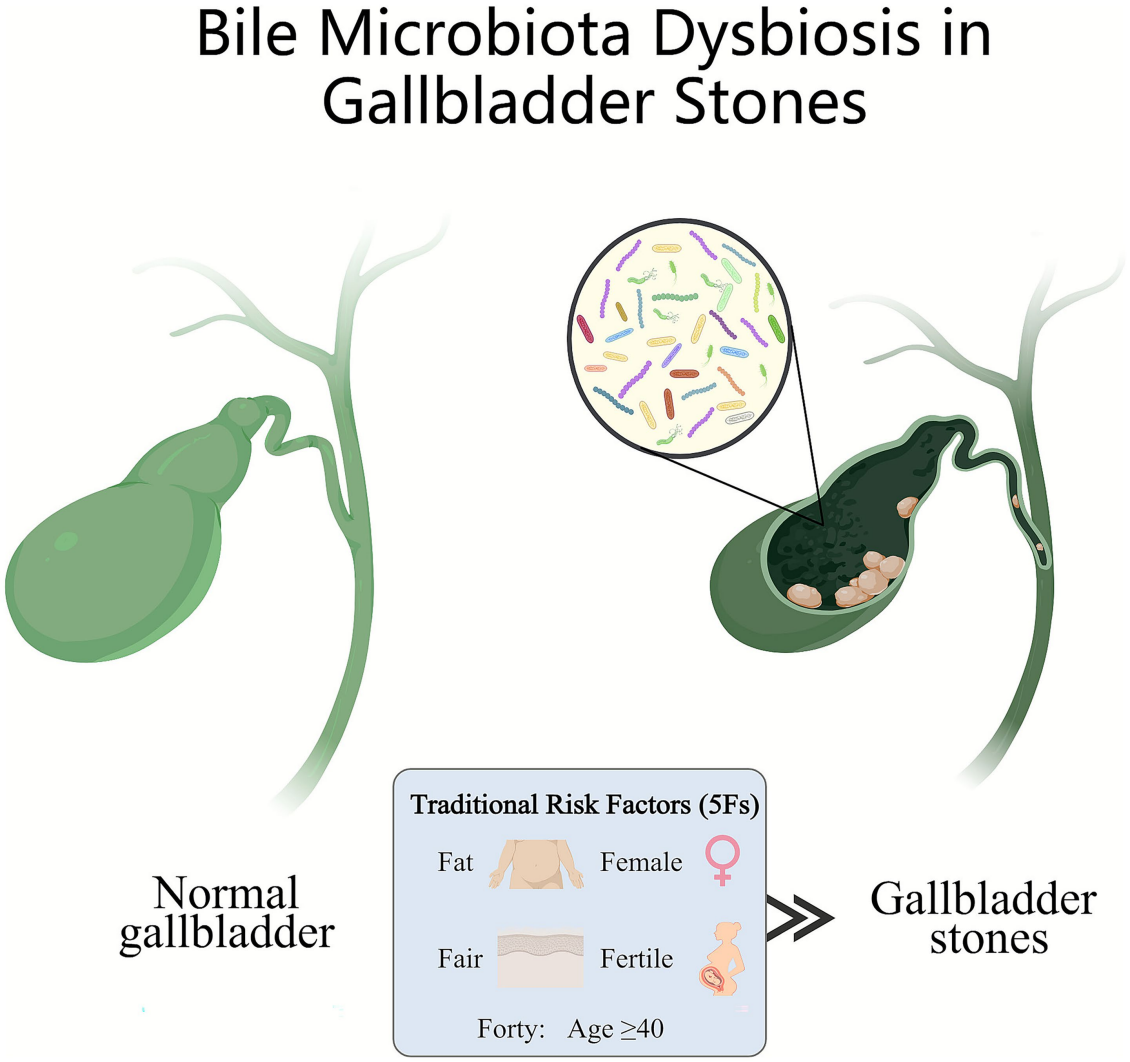
Bile microbiota dysbiosis in GS (created with MedPeer). The left panel shows a normal gallbladder with clear bile and a relatively balanced, low-biomass biliary microbiota. The right panel shows a gallbladder containing stones, with bile microbiota dysbiosis highlighted in the magnified inset. The box summarizes the “5Fs” for epidemiologic risk factors for GS—fat, female, fair, fertile, and forty (age ≥40 years), which are traditionally associated with an increased risk of GS.

This systematic review and meta-analysis evaluated quantitative and qualitative alterations of the microbiome in patients with GS compared to various control groups. GS was associated with a significant increase in microbial richness measured by the ACE index (Du et al., 2022; Lu, 2023; Park and Park, 2024; Wang, 2023), whereas no consistent changes were observed in the Shannon index (Cai et al., 2023; Du et al., 2022; Feng, 2021; Liao, 2021; Lu, 2023; Molinero et al., 2019; Park and Park, 2024; Wang, 2023). However, GS exhibited significantly increased *α*-diversity according to the Simpson index (Du et al., 2022; Feng, 2021; Liao, 2021; Lu, 2023; Park and Park, 2024; Wang, 2023). The *β*-diversity of gallbladder microbiota in patients with GS was significantly different from that of most control groups, although one study found no significant difference (Cai et al., 2023). At the phylum level, a consistent increase in Firmicutes and relative decrease in Proteobacteria were observed, whereas changes in Bacteroidetes, Actinobacteria, and Fusobacteria varied across studies (Cai et al., 2023; Du et al., 2022; Feng, 2021; Liao, 2021; Lu, 2023; Molinero et al., 2019; Park and Park, 2024; Wang, 2023). At the genus level, patients with GS exhibited increased abundance of taxa associated with potential pathogens, including *Escherichia-Shigella*, *Helicobacter,* and *Streptococcus* (Cai et al., 2023; Lu, 2023; Molinero et al., 2019; Park and Park, 2024; Yu et al., 2024a). These findings suggest that gallstone formation may be closely associated with bile microbiota dysregulation (Figure 4). In controls without hepatobiliary disease, 16S rRNA gene signals were detected in bile samples, indirectly suggesting that “healthy” bile may harbor microbiota; however, such findings should be interpreted cautiously because of the inherent risk of sample contamination.

### 4.1 Correlation between bile microbiota dysbiosis and GS

This is the first systematic review and meta-analysis to comprehensively search eight Chinese- and English-language databases and compare the bile microbiota between patients with GS and controls. Some studies have reported that patients with GS generally exhibited reduced α-diversity in their gut microbiota (Song et al., 2022), whereas this study indicated that the bile microbiota may display different patterns. This meta-analysis implies that patients with GS may exhibit alterations in bile microbial community structure, characterized by a trend toward increased species richness and overrepresentation of dominant taxa. This study indicated that GS-associated bile microbiota dysbiosis reflects a shift toward bile-tolerant taxa (Larabi et al., 2023). Bile constitutes a low-biomass, highly selective niche, which ordinarily constrains microbial colonization (Hazrah et al., 2004). Factors, such as genetic susceptibility, cholesterol supersaturation of gallbladder bile, impaired gallbladder emptying, rapid nucleation and growth of cholesterol crystals, and intestinal influences can remodel the gallbladder microenvironment (Sun et al., 2022). As available surfaces for adhesion expand and biofilms form, bile-tolerant taxa gain a competitive advantage within this niche (Bustos et al., 2018; Pumbwe et al., 2007). Moreover, community structure becomes uneven, with a few bile-tolerant taxa achieving functional dominance that may promote gallstone formation via multiple mechanisms. Current research has suggested that bacteria, such as *Escherichia coli* and *Pseudomonas aeruginosa,* secrete *β*-glucuronidase and phospholipase, which hydrolyze conjugated bilirubin into unconjugated bilirubin and lecithin into free fatty acids and lysophospholipids, thereby accelerating gallstone formation (Osnes et al., 1997; Peng et al., 2015; Zhang et al., 2025).

However, considering the lack of statistical significance in other richness metrics and indirect nature of diversity indices, further studies are warranted to determine whether microbial dysbiosis in GS is primarily driven by enrichment of dominant species rather than changes in overall richness. Additionally, the inconsistencies of phylum-level analysis suggested that shifts in the dominant phyla were not entirely uniform and may have been influenced by differences in control groups or study settings. This pattern differs from the gut microbiota composition associated with GS, although certain similarities are evident. The gut microbiota was dominated primarily by Firmicutes, followed by Bacteroidetes, Actinobacteria, and Proteobacteria (Dan et al., 2023). At the genus level, meta-analytic signals were generally weak. Patients with GS exhibited modestly increased abundance of facultative or environmental taxa, including *Lactobacillus*, *Escherichia–Shigella*, *Streptococcus*, and *Cupriavidus*, whereas genera, such as *Helicobacter,* were reported only in individual studies. These findings suggest that the GS-associated microbiome may be context-dependent rather than defined by a reproducible genus-level signature. Despite inter-study heterogeneity, recent work has substantiated specific pathogenic mechanisms within the biliary microenvironment. Yu et al. experimentally delineated this immunological pathway using human bile samples and mouse models, and showed that lipopolysaccharide (LPS) accumulation in bile activates the gallbladder mechanical barrier via the TLR4/MyD88/NF-κB axis. LPS also induces the release of neutrophil extracellular traps (NETs), thereby accelerating gallstone maturation (Yu et al., 2024b). Furthermore, Hu et al. employed a fecal microbiota transplantation (FMT) model and demonstrated that transfer of dysbiotic microbiota from gallstone patients to mice induces lithogenesis by modulating bile acid and cholesterol metabolism (Hu et al., 2022).

### 4.2 Controversial link between *Helicobacter pylori* and GS

Whether *H. pylori* infection is involved in the pathogenesis of GS remains inconclusive. A meta-analysis identified *H. pylori* colonization of the gallbladder as a significant risk factor for cholecystitis and cholelithiasis, with a stronger correlation observed in Asian populations. However, because of limitations in the design of the original studies, these findings suggested a statistical association rather than a definitive causal relationship (Cen et al., 2018). *H. pylori* may retrogradely migrate into the gallbladder via the stomach–duodenum–common bile duct pathway, thereby altering the local microenvironment (Lim et al., 2023). Lee et al. detected *H. pylori* DNA fragments in gallstones, bile, and gallbladder mucosa using PCR and sequencing techniques. However, the detection rates varied across different tissue types and were often confined to a single site, suggesting that *H. pylori* may be unable to establish stable colonization or exert sustained pathogenic effects within the gallbladder (Jin-Woo et al., 2010). In a subsequent investigation, among patients undergoing cholecystectomy, *H. pylori* was detected in the gastric mucosa but not in gallbladder tissue (Hegde et al., 2025). This observation implied *H. pylori* has a strong tropism for the gastric mucosa but only a limited ability to invade gallbladder tissue, thereby questioning its potential for long-term pathogenicity in the biliary system. Nonetheless, some mechanistic studies suggested that *H. pylori* may act as a co-promoting factor for gallstone formation under specific conditions. Yu et al. demonstrated that the bacterial virulence factor CagA downregulates the expression of tight junction proteins TJP1 and OCLN in gallbladder epithelial cells, leading to barrier disruption and increased permeability. In a mouse model of cholesterol gallstone formation, infection with CagA-positive strains significantly accelerated stone formation (Yu et al., 2024a). Moreover, the “bacterial nucleation hypothesis,” proposed in earlier studies, suggested that certain bacteria may serve as heterogeneous nucleation cores embedded within the developing gallstone, thereby promoting its formation. Supporting this concept, Lee et al. identified bacterial DNA in gallstone tissue (Jin-Woo et al., 2010). Belzer et al. showed that urease-positive *Helicobacter* species can induce calcium salt precipitation and purified urease alone exerts a similar effect, representing a potential mechanism in the formation of gallstones (Belzer et al., 2006).

The available evidence indicated an associative rather than causal relationship between *H. pylori* and GS. There was considerable heterogeneity across studies in populations, sampling sites, and detection methods, which further supports an associative, context-dependent link rather than a universal causal role. Therefore, we consider *H. pylori* unlikely to be a primary etiologic driver and more plausibly a context-dependent co-promoter in a susceptible biliary microenvironment. Future research should focus on strain-specific virulence factors, the integrity of the mucosal barrier, and the role of microbiota dysbiosis, preferably using prospective and interventional designs, to clarify the true etiological role of *H. pylori* in gallstone disease.

### 4.3 Limitations

Several limitations qualify our conclusions. First, the limited number of eligible studies and their relatively small sample sizes precluded funnel plot assessment and restricted the statistical power of additional subgroup analyses. Second, heterogeneity in DNA extraction protocols, 16S rRNA variable regions, and bioinformatic pipelines may have contributed to inflated variance. Third, all but one study originated from Asia, which limits the generalizability of findings to populations with distinct dietary and genetic backgrounds. Fourth, the effects of medications (proton-pump inhibitors and antibiotics) were inconsistently reported and not adjusted for, potentially confounding microbial signals. Fifth, functional inference based solely on 16S rRNA data remains speculative and requires confirmation through metagenomic and metabolomic profiling. Finally, further experimental validation using *in vivo* models is required to substantiate these associations.

## 5 Conclusion

This study demonstrated that GS was associated with alterations in the bile microbiota, characterized by increased microbial richness. *β*-diversity analyses revealed that patients with GS harbored distinct microbial communities compared with controls. At the phylum level, Firmicutes were consistently elevated, whereas Proteobacteria were decreased. At the genus level, including *Helicobacter*, patterns were heterogeneous and varied depending on study context or patient conditions. These findings suggest that microbial dysbiosis contributes to GS formation, which diverges from gut microbiota patterns traditionally associated with gallstone disease. The interpretation of these findings is limited by methodological heterogeneity, geographic concentration of studies, and reliance on 16S rRNA sequencing. Future multiregional studies integrating metagenomics, metabolomics, and experimental validation are needed to elucidate the causal mechanisms and functional roles of the bile microbiota in GS pathogenesis.

## Data Availability

The original contributions presented in the study are included in the article/Supplementary material, further inquiries can be directed to the corresponding author.

## Footnotes

1
https://www.math.hkbu.edu.hk/~tongt/papers/median2mean.html
