# Supplementary material for: Bile microbiota in gallbladder stones and its association with Helicobacter pylori: a systematic review and meta-analysis
Source: Front Microbiol. 2026 Jan 13;16:1707225. doi: 10.3389/fmicb.2025.1707225 (PMC12845325; doi:10.3389/fmicb.2025.1707225)

**Supplementary Material**

| **Database** | **PubMed** |
| --- | --- |
| Library: | Free access |
| Date of search | 3 July 2025 |
| Search query: | (((("Gallstones"[Mesh]) OR "Cholecystolithiasis"[Mesh]) OR "Cholelithiasis"[Mesh]) OR ((((((((((((((((Cholelithiases) OR (Gallstone Disease)) OR (Gallstone Diseases)) OR (Biliary Calculi)) OR (Calculi, Biliary)) OR (Gall Stone)) OR (Gall Stones)) OR (Gallstone)) OR (Common Bile Duct Calculi)) OR (Biliary Calculi, Common Bile Duct)) OR (Common Bile Duct Gall Stones)) OR (Common Bile Duct Gall Stone)) OR (Common Bile Duct Gallstones)) OR (Gall Stones, Common Bile Duct)) OR (Common Bile Duct Gallstone)) OR (Gallstones, Common Bile Duct))) AND (((("Microbiota"[Mesh]) OR "RNA, Ribosomal, 16S"[Mesh]) OR "Metagenome"[Mesh]) OR (((((((((((((((((((((((((Microbiotas) OR (Microbial Community)) OR (Community, Microbial)) OR (Microbial Communities)) OR (Microbial Community Composition)) OR (Community Composition, Microbial)) OR (Composition, Microbial Community)) OR (Microbial Community Compositions)) OR (Microbiome)) OR (Microbiomes)) OR (Human Microbiome)) OR (Human Microbiomes)) OR (Microbiome, Human)) OR (Microbial Community Structure)) OR (Community Structure, Microbial)) OR (Microbial Community Structures)) OR (Microflora)) OR (Flora)) OR (Metagenomes)) OR (16S)) OR (16S rRNA)) OR (rRNA, 16S)) OR (16S Ribosomal RNA)) OR (Ribosomal RNA, 16S)) OR (RNA, 16S Ribosomal))) |
| Number of results | 329 |

**Supplementary Table 1.** An example of the search strategy.

**Supplementary table 2.** NOS for the 9 studies

| Study | Selection | Comparability | Outcome | Total |
| --- | --- | --- | --- | --- |
| Yu et al., 2024a | ★★★ | ★ | ★ | 5 |
| Park et al., 2024 | ★★★★ | ★★ | ★★ | 8 |
| Cai et al., 2023 | ★★★ | ★★ | ★★ | 7 |
| Wang, 2023 | ★★★★ | ★★ | ★★ | 8 |
| Lu, 2023 | ★★★★ | ★★ | ★★ | 8 |
| Du et al., 2022 | ★★★★ | ★★ | ★★ | 8 |
| Liao, 2021 | ★★★★ | ★★ | ★★ | 8 |
| Feng, 2021 | ★★★★ | ★★ | ★★ | 8 |
| Molinero et al., 2019 | ★★★ | ★ | ★★ | 6 |

**Supplementary table 3.** RoBANS 2 for the 9 studies

| Study | Comparability  of the target  group | Target group  selection | Confounders | Measurement of  intervention/  exposure | Blinding of  assessors | Outcome  assessment | Incomplete  outcome  data | Selective  outcome  reporting |
| --- | --- | --- | --- | --- | --- | --- | --- | --- |
| Yu et al., 2024a | L | H | H | L | L | L | L | L |
| Park et al., 2024 | L | L | L | L | L | L | L | L |
| Cai et al., 2023 | L | L | L | L | L | L | L | L |
| Wang, 2023 | L | L | L | L | L | L | L | L |
| Lu, 2023 | L | L | L | L | L | L | L | L |
| Du et al., 2022 | L | L | L | L | L | L | L | L |
| Liao, 2021 | L | L | L | L | L | L | L | L |
| Feng, 2021 | L | L | L | L | L | L | L | L |
| Molinero et al., 2019 | L | L | L | L | L | L | L | L |

L, low risk; H, high risk.

**Supplementary table 4.** Genus-level differences in relative microbial abundance between gallstone patients and control groups

| Genus | Yu et al., 2024a | Park et al., 2024 | Cai et al., 2023 | Wang, 2023 | Lu, 2023 | Du et al., 2022 | Liao, 2021 | Feng, 2021 | Molinero et al., 2019 |
| --- | --- | --- | --- | --- | --- | --- | --- | --- | --- |
| Statistical differences | **+** | **-** | **-** | **+** | **-** | **+** | **+** | **+** | **+** |
| *Helicobacter* | Higher （G） |  |  |  |  |  |  |  |  |
| *Salmonella* |  |  |  | Higher (C) |  |  |  |  |  |
| *Faecalibacterium* |  |  |  | Higher (G) |  |  |  |  |  |
| *Sphingomonas* |  |  |  |  | Higher (C) |  |  |  | Higher (C) |
| *Bradyrhizobium* |  |  |  |  | Higher (C) |  |  |  | Higher (C) |
| *Pseudomonas* |  |  | Higher (C) |  | Higher (C) |  |  |  |  |
| *Ralstonia* |  | Higher (G) | Higher (C) |  | Higher (C) |  |  | Higher (G) |  |
| *Bacteroides* |  |  |  |  | Higher (C) |  |  |  | Higher (G) |
| *Acidibacter* |  |  |  |  | Higher (C) |  |  |  | Higher (C) |
| *Vibrio* |  |  | Higher (C) |  | Higher (G) |  |  |  |  |
| *Cupriavidus* |  |  |  |  | Higher (G) |  |  | Higher (G) |  |
| *Thauera* |  |  |  |  | Higher (G) |  |  |  |  |
| *Streptococcus* |  | Higher (G) |  |  | Higher (G) |  |  |  |  |
| *Roseburia* |  |  |  |  |  | Higher (C) |  |  |  |
| *Lactobacillus* |  | Higher (G) | Higher (G) |  |  |  |  |  |  |
| *Enterococcus* |  | Higher (G) |  |  |  |  |  |  |  |
| *Escherichia-Shigella* |  |  | Higher (G) |  |  |  |  | Higher (C) | Higher (G) |
| *Neisseria* |  |  | Higher (G) |  |  |  |  |  |  |
| *Stenotrophomonas* |  |  | Higher (G) |  |  |  |  |  |  |
| *Lachnoanerobaculum* |  |  | Higher (G) |  |  |  |  |  |  |
| *Atopobium* |  |  | Higher (G) |  |  |  |  |  |  |
| *Oribacterium* |  |  | Higher (G) |  |  |  |  |  |  |
| *Peptostreptococcus* |  |  | Higher (G) |  |  |  |  |  |  |
| *Rothia* |  |  | Higher (G) |  |  |  |  |  |  |
| *Stomatobaculum* |  |  | Higher (G) |  |  |  |  |  |  |
| *Klebsiella* |  |  | Higher (G) |  |  |  |  |  |  |
| *Prevotella* |  |  | Higher (G) |  |  |  |  |  |  |
| *Serratia* |  |  | Higher (G) |  |  |  |  |  |  |
| *Veillonella* |  |  | Higher (G) |  |  |  |  |  |  |
| *Pseudoalteromonas* |  |  | Higher (C) |  |  |  |  |  |  |
| *Providencia* |  |  | Higher (C) |  |  |  |  |  |  |
| *Parapusillimonas* |  |  | Higher (C) |  |  |  |  |  |  |
| *Paracoccus* |  |  | Higher (C) |  |  |  |  |  |  |
| *Ochrobactrum* |  |  | Higher (C) |  |  |  |  |  |  |
| *Lachnoanaerobaculum* |  |  | Higher (G) |  |  |  |  |  |  |
| *Diaphorobacter* |  |  | Higher (C) |  |  |  |  |  |  |
| *Comamonas* |  |  | Higher (C) |  |  |  |  | Higher (G) |  |
| *Citrobacter* |  |  | Higher (C) |  |  |  |  |  |  |
| *Castellaniella* |  |  | Higher (C) |  |  |  |  |  |  |
| *Brevundimonas* |  |  | Higher (C) |  |  |  |  |  | Higher (C) |
| *Bordetella* |  |  | Higher (C) |  |  |  |  |  |  |
| *Dialister* |  |  |  |  |  |  |  |  | Higher (G) |
| *Methylobacterium* |  |  |  |  |  |  |  |  | Higher (C) |
| *Acinetobacter* |  |  |  |  |  |  |  | Higher (G) |  |

G, patients with gallstone; C, control group. When statistical differences are denoted by “+” in a study, the list includes only those genera with statistically significant differences. When denoted by “–”, the genera are qualitatively compared according to the descriptions and figures provided in the study.


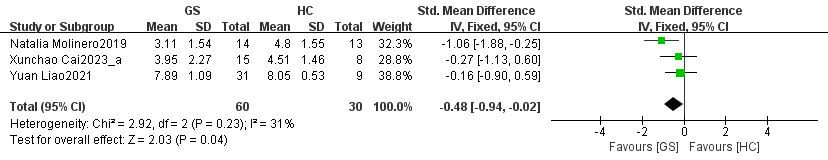
**Supplementary Figure 1.** Shannon Index between gallbladder stones (GS) and healthy controls (HC) by fixed-effects model

A


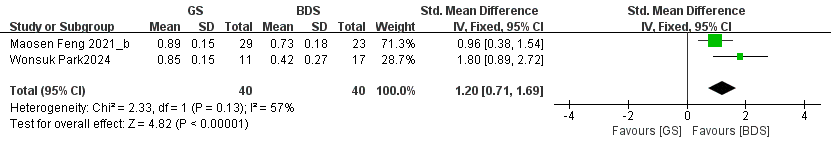


**Supplementary Figure 2.** A.Simpson index between GS and bile duct stones (BDS) by fixed-effects model; B.Simpson index between GS and other controls (e.g., cholecystitis, gallbladder polyps) by fixed-effects model

B


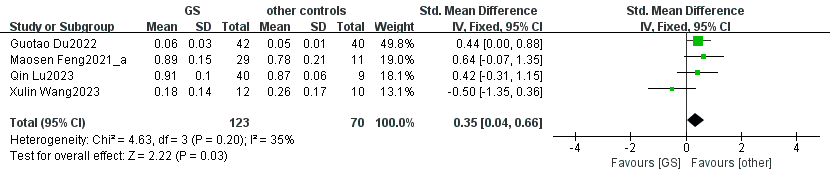

Supplement: Supplementary file 1 [file Data_Sheet_1.DOCX]
